# Assessing the performance of a portable electroencephalographic sleep monitor against level 1 polysomnography

**DOI:** 10.1093/sleepadvances/zpaf089

**Published:** 2025-12-12

**Authors:** Malika Lanthier, Michael-Christopher Foti, Karina Fonseca, Caitlin Higginson, Defne Oksit, David Smith, Jean-Marc Lina, Paniz Tavakoli, Stuart Fogel, Laura Ray, Rébecca Robillard, Canadian Sleep Research Consortium, Malika Lanthier, Micheal-Christopher Foti, Karina Fonseca, Caitlin Higginson, Defne Oksit, David Smith, Jean-Marc Lina, Paniz Tavakoli, Stuart Fogel, Laura Ray, Rébecca Robillard

**Affiliations:** Sleep Research Unit, University of Ottawa Institute of Mental Health Research at the Royal, Ottawa, ON K1Z 7K4, Canada; School of Psychology, University of Ottawa, Ottawa, ON K1N 6N5, Canada; Sleep Disorders Clinic, Royal Ottawa Mental Health Centre, Ottawa, ON K1Z 7K4, Canada; Sleep Research Unit, University of Ottawa Institute of Mental Health Research at the Royal, Ottawa, ON K1Z 7K4, Canada; School of Psychology, University of Ottawa, Ottawa, ON K1N 6N5, Canada; Sleep Research Unit, University of Ottawa Institute of Mental Health Research at the Royal, Ottawa, ON K1Z 7K4, Canada; Sleep Disorders Clinic, Royal Ottawa Mental Health Centre, Ottawa, ON K1Z 7K4, Canada; Sleep Research Unit, University of Ottawa Institute of Mental Health Research at the Royal, Ottawa, ON K1Z 7K4, Canada; Sleep Research Unit, University of Ottawa Institute of Mental Health Research at the Royal, Ottawa, ON K1Z 7K4, Canada; Department of Cellular and Molecular Medicine, University of Ottawa, Ottawa, ON K1H 8M5, Canada; Département de Génie Électrique, École de Technologie Supérieure, Montréal, QC H3C 1K3, Canada; Sleep Research Unit, University of Ottawa Institute of Mental Health Research at the Royal, Ottawa, ON K1Z 7K4, Canada; Sleep Research Unit, University of Ottawa Institute of Mental Health Research at the Royal, Ottawa, ON K1Z 7K4, Canada; School of Psychology, University of Ottawa, Ottawa, ON K1N 6N5, Canada; School of Psychology, University of Ottawa, Ottawa, ON K1N 6N5, Canada; Sleep Research Unit, University of Ottawa Institute of Mental Health Research at the Royal, Ottawa, ON K1Z 7K4, Canada; School of Psychology, University of Ottawa, Ottawa, ON K1N 6N5, Canada

**Keywords:** portable electroencephalography, polysomnography, electrophysiology, instrumentation, scoring, performance assessment, validation, sleep/wake physiology, EEG analysis, home testing

## Abstract

**Study Objectives:**

To assess the performance of a portable electroencephalography device for sleep monitoring against polysomnography.

**Method:**

Fifty-six adults underwent one night of in-laboratory sleep recording with the Muse-S headband and simultaneous level 1 polysomnography. Muse-S data were scored by an automated sleep staging algorithm. A registered technologist, blind to the Muse-S automated sleep scoring, scored the polysomnography data.

**Results:**

Good quality data were available for 47 (84 per cent) participants (53 per cent females; 20–71 years old; 17 per cent with sleep-related breathing disorder). Epoch-by-epoch analyses showed substantial agreement between the Muse-S and polysomnography (full night Cohen’s Kappa = 0.76). Cohen’s Kappa were in the fair agreement range for non-rapid eye movement (NREM) 1, substantial agreement range for NREM2 and NREM3, and near-perfect agreement range for rapid eye movement sleep and wake. Accuracy ranged from 88 per cent to 96 per cent across all sleep stages, with a sensitivity of 79–92 per cent and a specificity of 90–99 per cent. Similar results were observed in the subgroup with sleep-related breathing disorder. On average, the Muse-S had higher mean values than polysomnography for total sleep time (+6 min), NREM3 (+15 min), rapid eye movement sleep (+6 min), and sleep efficiency (+1.5 per cent), and lower mean values for sleep latency (−3 min), wake after sleep onset (−3 min), and light sleep (−14 min).

**Conclusions:**

When compared to standard polysomnography, the Muse-S performed well to measure sleep macroarchitecture. This portable device shows great potential as an accessible tool for sleep electroencephalography monitoring. More work is required to validate this tool in more diverse populations to ensure robustness across age, sex, neurological conditions, and sleep profiles.

This article is part of the Consumer Sleep Technology Special Collection

**Statement of Significance**

Most portable monitors are restricted to indirect measures to estimate sleep. This study offers an independent assessment of the performance of a portable electroencephalography headband. Compared to in-laboratory polysomnography, this type of device enables more accessible multi-night data collection in the natural sleeping environment. Such technologies have tremendous potential to expand research capacity and clinical applications. This article may help to inform the choice of appropriate technologies to be used to address specific research questions and to anticipate the strengths and limitations of this new technology.

## Introduction

Portable sleep trackers are increasingly being used by consumers, researchers, and clinicians to measure sleep–wake states longitudinally outside of the laboratory [1]. To this date, home-based sleep monitoring has mostly been done with wrist-worn accelerometry sensors, which estimate sleep based on movement. This has shown to be very helpful to delineate sleep–wake profiles over multiple nights in the field. However, these devices hold major limitations. Firstly, they only provide indirect estimations of sleep and wake states as they do not enable direct measurements of brain activity (i.e. electroencephalography [EEG]). Secondly, the sleep estimates generated by these devices are restricted to basic sleep metrics. These metrics are typically derived from the succession of each epoch of recording being scored as sleep or wake (i.e. binary) and remain blind to sleep stages and features of sleep microarchitecture (e.g. spindles, slow oscillations, rapid eye movements, and spectral power across different frequency bands).

Sleep is a complex physiological state regulated by intricate interactions between the central nervous system and peripheral mechanisms. The accurate characterization of sleep architecture, including different depths of non-rapid eye movement (NREM) sleep, rapid eye movement (REM) sleep, and the intrusion of periods of stages of wakefulness, is essential for in-depth sleep monitoring. Polysomnography, the gold standard of sleep monitoring, entails the simultaneous recording of multiple physiological signals, including EEG, electromyography (EMG), and electrooculography (EOG), to classify sleep stages following standardized scoring criteria such as those outlined by the American Academy of Sleep Medicine (AASM). However, the accessibility of polysomnography is limited by the need for specialized equipment, trained personnel, high costs, and artificial sleep environment imposed by laboratory settings.

More recently, portable EEG devices have emerged as promising new tools for more accessible and scalable in-depth sleep monitoring. These devices offer the potential to democratize detailed sleep assessments, enabling widespread, cost-effective, and longitudinal studies that were previously constrained by the logistical and financial burdens of in-laboratory polysomnography. Portable EEG devices typically integrate lightweight, user-friendly designs with automated signal processing algorithms to classify sleep stages. They are often restricted to a limited number of channels utilizing dry electrodes to enhance comfort and ease of use. Such trade-offs necessitate rigorous performance assessment to ensure that data from these devices accurately reflect sleep patterns and physiological parameters.

A systematic review assessing over 42 studies evaluating the performance of 24 different portable EEG devices highlighted considerable variability across studies in terms of accuracy (ranging from 0.48 to 0.95) and specificity (ranging from 0.11 to 0.98) [2]. There was also variability across sleep stages, with NREM1 sleep being the most difficult sleep stage to identify correctly with these devices. A new portable EEG device available on the consumer market, the Muse-S headband (InteraXon, Toronto, Canada), was initially developed to support meditation biofeedback and has been used to measure drowsiness, and event-related potentials [3], but holds great potential for sleep monitoring. The Muse-S records brain activity with dry electrodes, heart rate with a photoplethysmography sensor as well as breathing and body movement with an accelerometer and a gyroscope. Sleep macroarchitecture and spectral power metrics are automatically measured by InteraXon’s sleep staging algorithm, which has been validated against two expert registered polysomnographic technologists [4].

The present study aimed to assess the performance of the Muse-S device against simultaneous polysomnography recording. Specifically, global sleep architecture parameters and epoch-to-epoch sleep stage scoring derived from the automated Muse-S scoring algorithm were compared to polysomnography data scored by an expert registered polysomnographic technologist. A secondary aim was to get a sense of the level of agreement between the Muse-S device and standard polysomnography in people with sleep-related breathing disorder.

## Materials and Methods

### Participants

Fifty-six male and female adults completed this study. To maximize diversity, no selection criteria were used aside from the ability to provide informed consent.

Written informed consent was obtained from all participants. The University of Ottawa’s Health and Sciences Research Ethics Board and the Human Ethics Board of the Royal Ottawa Health Care Group approved the study. The study was conducted according to the Declaration of Helsinki [5].

### Procedures

All participants completed questionnaires to document demographic, sleep, and mental health characteristics for descriptive purposes. This included the Pittsburgh Sleep Quality Index (PSQI) [5], the reduced morningness-eveningness questionnaire [6], and the Beck's Depression Inventory-II [7].

Participants then underwent an overnight sleep recording in the laboratory according to their habitual sleep–wake schedules with the Muse-S device and simultaneous level 1 polysomnography. Level 1 polysomnography was used as the gold-standard measure.

#### Standard polysomnography

Standard level 1 polysomnography was recorded with the Embla Communication Unit or M Drive amplifiers, using the N7000 headbox and REMLogic software (Natus, Kanata, ON). Data were acquired at a sampling rate of 256 Hz. Electrodes were placed according to the 10–20 system with a minimum of 3 EEG channels (F3-M2, C3-M2, and O1-M2), a ground and reference (Fpz), right and left EOG, two chin and four leg EMG, and two electrocardiogram channels.

#### Recordings with the portable EEG monitor

**Figure 1:**
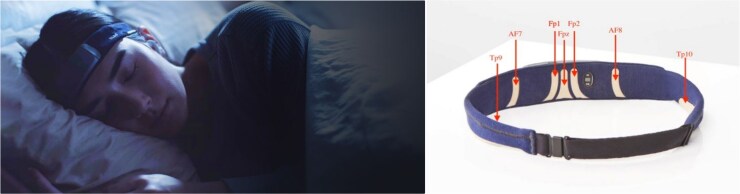
Visual display of Muse-S and electrode placement configurations.

The Muse-S, a soft wireless headband with dry electrodes, was placed around the participant’s head in addition to standard polysomnography EEG set-up. The Muse-S has four EEG channels (Fp1, Fp2, Tp9, Tp10) recording at a sampling rate of 256 Hz, a three-axis accelerometer with a sampling rate of 52 Hz, and a photoplethysmography sensor (see Figure 1 for visual representation). Signals are combined in four derivations referenced to Fpz: Fp1–Fpz, Fp2–Fpz, Tp9–Fpz, and Tp10–Fpz. Data collected from the Muse-S were sent from the headband to an adjacent iPad via Bluetooth. In the morning, after data collection was completed, data were transferred from the iPad to a data processing cloud via Wi-Fi.

### Data processing and computation of sleep architecture parameters

Prior to data processing, the Muse-S data were manually aligned with the timestamps of “lights off” and “lights on” event markers from level 1 polysomnography to determine the delimitation of the 30-s epochs of recording to be scored. This temporal alignment was confirmed using the timing of specific sequences of eye blinks that occurred during the biocalibration prior to “lights off.” Signal quality assessments for each Muse-S channel and automated sleep stage scoring for Muse-S data were completed using automated algorithms developed by InteraXon (sleep staging v2). InteraXon remained blinded to all components of level 1 polysomnography data, except for the “lights off” and “lights on” timestamps that were used for temporal alignment.

One registered sleep technologist manually scored all the standard polysomnography recordings in 30-s epochs according to guidelines established by the American Academy of Sleep Medicine (v3.0) [8]. This technologist was blind to the automated Muse-S sleep stage scoring.

Equivalent sleep architecture parameters were computed based on human-scored polysomnography and automated sleep scoring of the Muse-S data. This included: total sleep time, sleep onset latency, wake after sleep onset, sleep efficiency, and relative and absolute time spent in each sleep stages (NREM1, NREM2, NREM3, REM). Sleep onset latency was defined as the time elapsed between lights off and the first epoch of sleep. Total sleep time was defined as the time spent asleep between the “lights off” and “lights on” markers. Sleep efficiency was computed as total sleep time divided by time in bed multiplied by 100. Absolute sleep stages were calculated as the total number of minutes spent in NREM1, NREM2, NREM3, and REM sleep. Relative sleep stages were calculated as the percentage of time between the “lights off” and “lights on” markers scored as a given sleep stage divided by total sleep time.

### Statistical analyses

Sleep metrics collected with the Muse-S and polysomnography were compared using paired *t*-tests. Wilcoxon Signed Ranks Tests were used for variables that were not normally distributed based on Kolmogorov–Smirnov tests of normality (this was the case for sleep onset latency, sleep efficiency, wake after sleep onset, the number of minutes and percentage of deep sleep and the percentage of REM sleep). We also calculated the proportions of recordings falling within thresholds for satisfactory differences proposed in previous studies [9–11]: ≤30 min for total sleep time and wake after sleep onset, and <5 per cent for sleep efficiency. Since time in bed for the Muse-S device was aligned to the “lights off” and “lights on” polysomnography markers, this parameter was not compared across both sets of recordings.

Based on epoch-by-epoch comparisons of Muse-S versus polysomnography, we calculated Cohen’s Kappa coefficient of agreement [12] for all epochs contained between the “lights off” and “lights on” markers, as well as for all epochs of wake and all sleep stages separately. Interpretation of Kappa values were based on the following thresholds: 0–0.20 slight agreement, 0.21–0.40 fair agreement, 0.41–0.60 moderate agreement, 0.61–0.80 substantial agreement, and 0.81–1.00 almost perfect agreement [13]. Furthermore, for wake, light sleep (NREM1 + NREM2), deep sleep (NREM3), and REM sleep, accuracy, sensitivity, specificity metrics were computed, receiver operating curves were produced to contrast true positive (sensitivity) and false positive rates (1-specificity), and Bland–Altman plots were used to assess measurement agreement across levels of standard polysomnography parameters. Specifically, sensitivity was calculated as the proportion of epochs classified as a given sleep stage by level 1 polysomnography that were correctly classified by the Muse-S device. Specificity was calculated as the proportion of epochs not classified as a given sleep stage by level 1 polysomnography that were correctly not classified as that stage by the Muse-S device. Accuracy was calculated as the proportion of correctly classified epochs for a given sleep stage over the total number of epochs. Secondary analyses were conducted to derive the same validation metrics in a subgroup with sleep-related breathing disorder objectified through level 1 polysomnography based on an apnea-hypopnea index (AHI) *>* 5.

All analyses were done in MATLAB [14] and notably leveraged an existing open-source code for testing the performance of sleep tracking technologies [15].

## Results

### Sample characteristics

Of the 56 recordings, nine (16 per cent) were excluded because of poor signal quality on the Muse-S device. Full sample characteristics and sleep profiles for the remaining 47 participants are reported in Table 1. In brief, ages ranged from 20 to 71 years old (mean (SD) = 31.7 (13.3) years old) and the sample counted 25 (53 per cent) females. Twenty five per cent of the final sample had taken a sleep medication in the past month. In the month prior to the study, self-reported usual bedtime ranged from 8.30 pm to 4 am, and wake up time ranged from 4.30 am to 1 pm, with an estimated sleep duration between 4 and 9 h. In terms of chronotypes, 17 per cent were moderate morning types and 28 per cent were either moderate or definite evening types. PSQI scores ranged from 1 to 15, with 80 per cent of the sample having a score above the cut-off for significant sleep disturbances. Eight (17 per cent) participants had sleep-related breathing disorder objectified through level 1 polysomnography scored by registered sleep technologists (AHI ranging from 5.2 to 52.4; mean = 17.0, SD = 17.8).

**Table 1:** Sample characteristics

Characteristics	%	Min	Max	Mean	SD
Age (years)		20	71	31.7	13.3
Females (%)	53%	-	-	-	-
Sleep medications (%)	25%	-	-	-	-
Usual bedtime		20:30	4:00	23:24	1:30
Usual wake up time		4:30	13:00	7:36	1:36
Usual total sleep time (min)		240	540	396	72
PSQI total score		1	15	7.0	3.2
rMEQ total score		6	20	13.7	3.5
Chronotypes					
Definite evening	3%	-	-	-	-
Moderate evening	25%	-	-	-	-
Neither	55%	-	-	-	-
Moderate morning	17%	-	-	-	-
Definite morning	0%	-	-	-	-

Min: Minimum, Max: Maximum, SD: Standard Deviation. Sleep medications: used at least once per week in the past month; self-reported typical bedtime, wake up time, and total sleep time for the past month; PSQI: Pittsburgh Sleep Quality Index; rMEQ: reduced morningness-eveningness questionnaire.

### Visual data inspection

**Figure 2:**
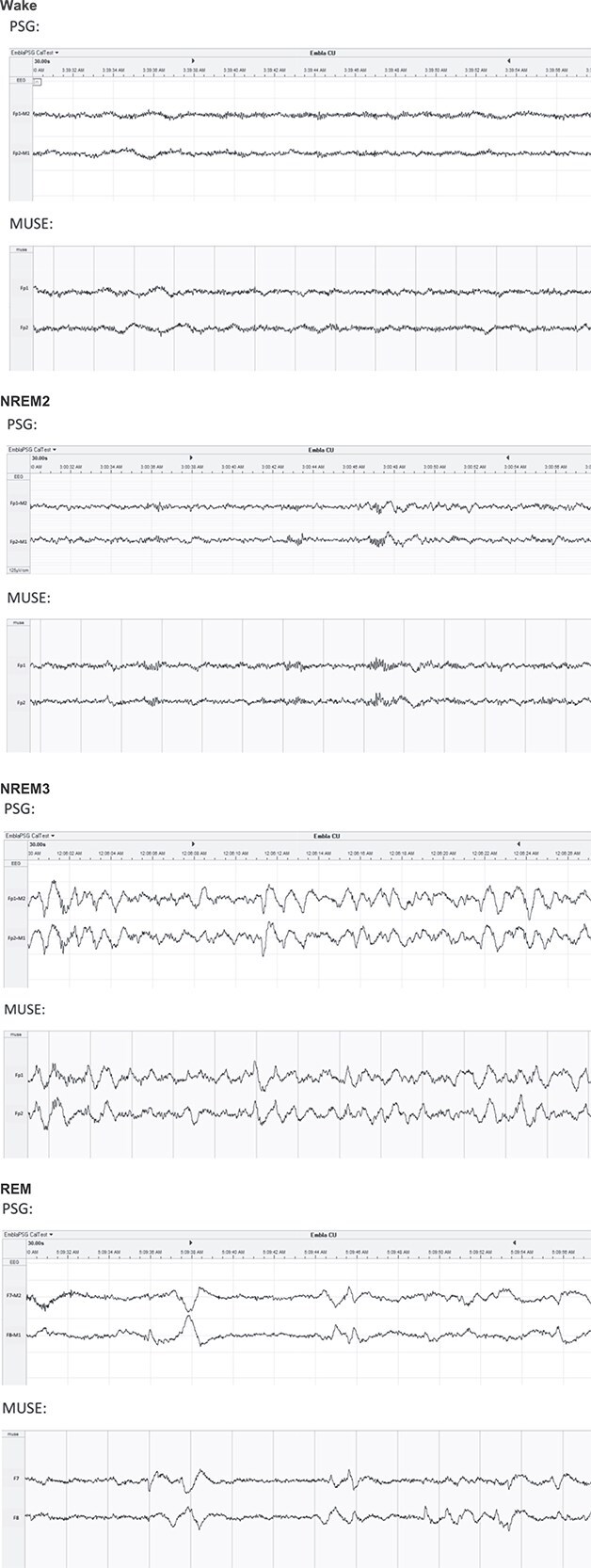
Visual display of simultaneous Muse-S and polysomnography signals. Examples of Muse-S and polysomnography recordings collected for the most proximal electrodes using the same 30-s epochs in wake, NREM2, NREM3, and REM sleep.

Figure 2 presents examples of simultaneous Muse-S and polysomnography recordings collected for the same 30-s epochs. Visual inspection of the raw signals revealed that microarchitecture elements, such as *k*-complexes, sleep spindles, and slow waves, that occurred on the standard polysomnography recording were also visible from the EEG signals recorded with the Muse-S device.

### Epoch-by-epoch concordance

Epoch-by-epoch analyses for all sleep stages combined showed that the Muse-S device reached substantial agreement with level 1 polysomnography, as reflected by a Cohen’s Kappa of 0.76 (CI: 0.75–0.76). As can be seen in Table 2, finer grained analyses revealed some variability across distinct sleep stages. Cohen’s Kappa scores were in the fair to moderate agreement range for NREM1 sleep, increased to the substantial agreement range for both NREM2 and NREM3 sleep, and further increased to the near-perfect agreement range for REM sleep and wake.

**Table 2:** Epoch-by-epoch concordance

Stage	Cohen's Kappa	95% CI
All epochs	0.76	0.75–0.76
Wake	0.84	0.83–0.84
NREM1	0.41	0.39–0.43
NREM2	0.75	0.74–0.75
NREM3	0.77	0.76–0.77
REM	0.85	0.85–0.86

95% CI: 95% confidence interval of Cohen’s Kappa.

**Figure 3:**
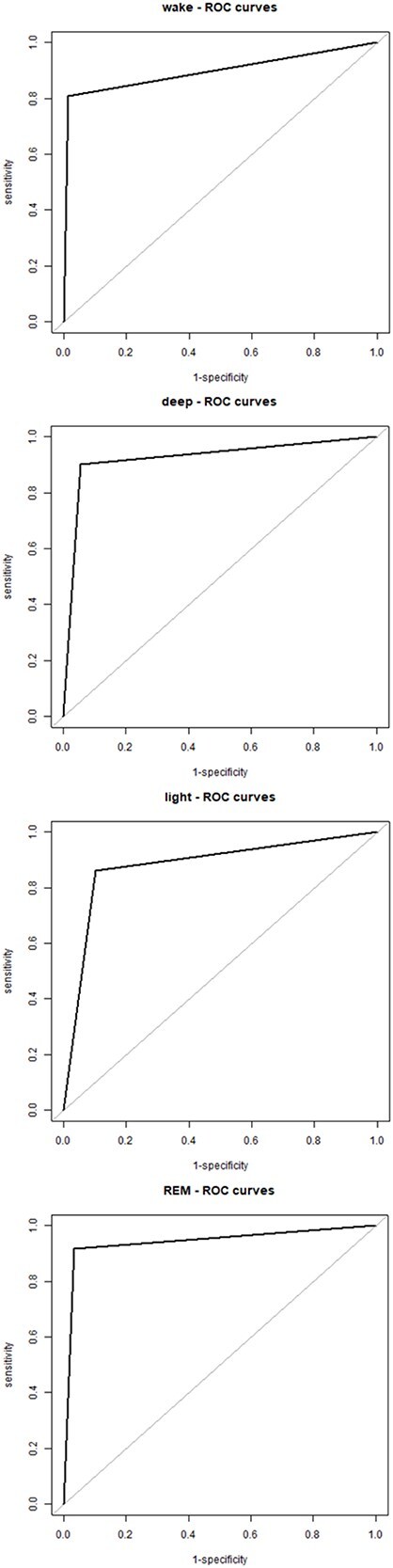
Receiver operating characteristic (ROC) curve. ROC curve showing the sensitivity and specificity of sleep stages identified by the Muse-S device as compared to level 1 polysomnography for wake, light sleep (i.e. NREM1 and NREM2), deep sleep (i.e. NREM3), and REM sleep.

The accuracy of the Muse-S relative to standard polysomnography ranged between 88 percent and 96 percent across all sleep stages, with a sensitivity of 79 percent to 92 percent, and a specificity of 90 percent to 99 percent (Table 3). Receiver operating characteristic curve revealed a good trade-off between sensitivity and specificity (Fig. 3).

**Table 3:** Accuracy, sensitivity, and specificity of the Muse-S relative to standard polysomnography

	Accuracy				Sensitivity				Specificity			
			95% CI				95% CI				95% CI	
Stage	Mean	SD	Lower	Upper	Mean	SD	Lower	Upper	Mean	SD	Lower	Upper
Wake	96.4	2.2	95.8	97.0	78.9	12.7	75.3	82.7	98.7	1.1	98.4	99.0
Light sleep	87.7	4.3	86.5	88.9	85.9	7.3	83.9	88.0	90.0	5.0	88.6	91.5
Deep sleep	93.8	3.8	92.8	94.9	88.3	17.1	84.1	93.8	94.3	5.1	92.9	95.8
REM sleep	96.0	2.3	95.3	96.6	91.5	8.0	89.4	93.9	96.8	2.5	96.1	97.5

95% CI: 95% confidence intervals.

**Figure 4:**
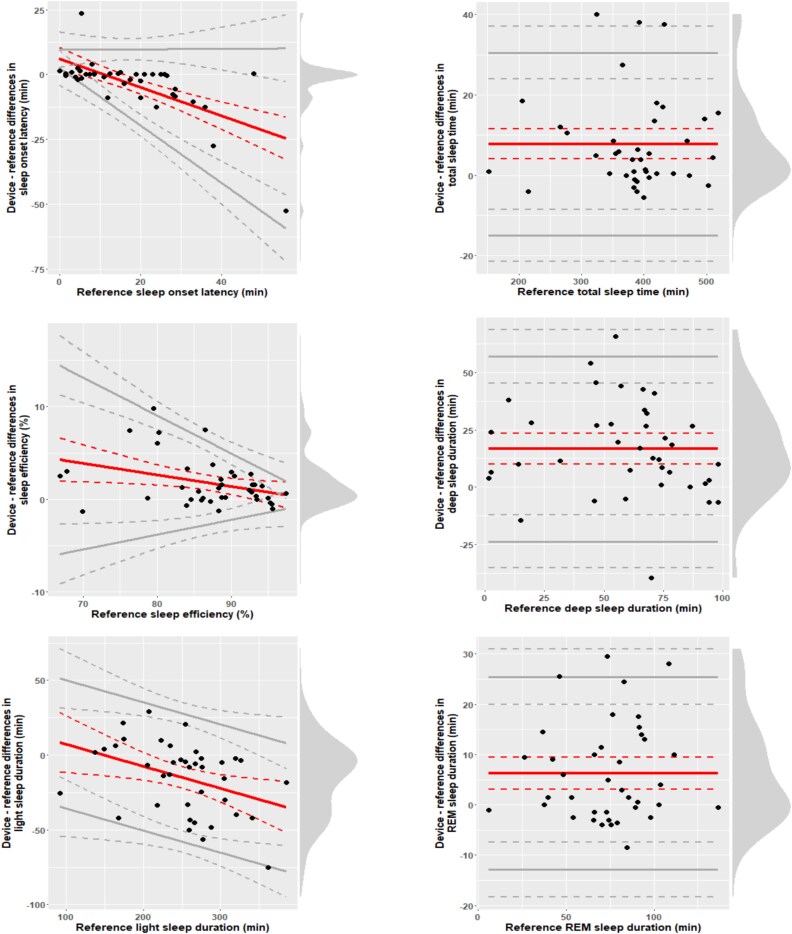
Bland–Altman plots. Central (red) solid lines indicate bias, peripheral (gray) solid lines indicate the 95% limits of agreement (LOAs), dotted lines indicate 95% CIs. Dots indicate individual observations. Vertical density diagrams on the right side of each plot represent the distribution of the differences. Plots are adjusted for the specific case of compliance with the assumptions for discrepancy analysis: All-fulfilled (total sleep time, “deep” sleep duration, and REM sleep duration), proportional bias and homoscedastic differences (“light” sleep duration), proportional bias, and heteroscedastic differences (sleep onset latency and sleep efficiency).

Figure 4 presents Bland–Altman plots for core sleep architecture parameters. More pronounced biases in Muse-S measures of sleep onset latency, sleep efficiency, and light sleep were, respectively, associated with longer sleep onset latencies, poorer sleep efficiency, and larger amounts of light sleep. Muse-S biases in sleep onset latency and sleep efficiency also became more variable for longer sleep onset latencies and shorter sleep efficiency values. Muse-S biases were more stable across different levels of total sleep time, deep sleep, and REM sleep.

### Overall comparisons between Muse-S and polysomnography data

Table 4 reports means and standard deviations for sleep architecture parameters measured by standard polysomnography and the Muse-S device. Overall, statistically significant differences were found for all parameters, with small-to-medium effect sizes except for the percentage of light sleep, for which a large effect size was found. Compared to standard polysomnography, the Muse-S had higher mean values for total sleep time (by 6.7 min), sleep efficiency (by 1.5 per cent), the amount of deep sleep and REM sleep (by 15.2 and 6.4 min, respectively), and the percentages of deep sleep and REM sleep (by 3.8 per cent and 1.3%, respectively). Conversely, compared to standard polysomnography, the Muse-S had lower mean values for sleep onset latency and wake after sleep onset (by 3.4 and 3.3 min, respectively), and light sleep (by 14.9 min and 5.1 per cent). Based on established thresholds, 93.6 per cent of recordings fell within the satisfactory difference range for total sleep time, 91.5 per cent for sleep efficiency, and 97.9 per cent for wake after sleep onset.

**Table 4:** Sleep parameters measured by standard polysomnography and the Muse-S

	Polysomnography		Muse		Bias				Cohen's *d*	95% CI	
Parameters	Mean	SD	Mean	SD	Mean	SD	*F/Z*	*P*		Lower	Upper
TST (min)	386.3	75.3	393.0	76.0	6.7	11.0	−4.2	<.001	−0.62	−0.92	−0.30
Sleep efficiency (%)	86.3	9.4	87.8	9.0	1.5	2.4	−3.9	<.001	−0.61	−0.92	−0.30
SOL (min)	17.8	17.0	14.4	11.2	−3.4	11.4	−2.2	.027	0.30	0.00	0.59
WASO (min)	43.8	33.8	40.5	35.2	−3.3	11.0	−2.1	.032	0.31	0.01	0.60
Light sleep (min)	253.1	57.2	238.2	54.8	−14.9	22.8	4.5	<.001	0.65	0.34	0.97
Deep sleep (min)	59.8	25.2	75.0	30.0	15.2	20.6	−4.3	<.001	−0.74	−1.06	−0.41
REM sleep (min)	73.4	29.2	79.8	29.1	6.4	11.7	−3.8	<.001	−0.55	−0.86	−0.24
Light sleep (%)	65.6	8.5	60.5	8.0	−5.1	6.1	5.8	<.001	0.84	0.51	1.17
Deep sleep (%)	16.0	7.7	19.8	9.4	3.8	5.9	−4.0	<.001	−0.65	−0.96	−0.33
REM sleep (%)	18.4	5.6	19.7	5.2	1.3	3.0	−2.2	.030	−0.43	−0.73	−0.13

TST: total sleep time, SOL: sleep onset latency, WASO: wake after sleep onset, 95% CI: 95% confidence interval of paired sample Cohen’s *d*, SD: standard deviation.

### Exploratory analyses in the subgroup with sleep-related breathing disorder

As can be seen in Table 5, the agreement between the Muse-S and polysomnography for all sleep stages in the subgroup with sleep-related breathing disorders was very close (Cohen’s Kappa of 0.74, CI: 0.73–0.75) to that observed in the full sample (Cohen’s Kappa of 0.76, CI: 0.75–0.76). A similar pattern was also found in the agreements for different sleep stages, with the lowest level of agreement for NREM1 sleep.

**Table 5:** Epoch-by-epoch concordance for the subgroup with sleep apnea

Stage	Cohen's Kappa	95% CI
All epochs	0.74	0.73–0.75
Wake	0.87	0.85–0.88
NREM1	0.32	0.28–0.38
NREM2	0.71	0.70–0.73
NREM3	0.81	0.79–0.83
REM	0.80	0.78–0.82

95% CI: 95% confidence interval of Cohen’s Kappa.

## Discussion

This study provides an independent assessment of the performance of a portable EEG device contrasted to gold-standard polysomnography, shedding light on its capabilities and limitations as a tool for sleep monitoring. Overall, the findings show that the Muse-S and its automated scoring algorithm hold substantial agreement with human-scored standard polysomnography. This was reflected in the comparison of global sleep architecture parameters and epoch-by-epoch sleep stage classification metrics. These results confirm the strong performance of the Muse-S system for measuring sleep macroarchitecture. This technology holds promise as a portable, cost-effective tool for EEG-based sleep monitoring.

The Muse-S device exhibited strong performance in measuring global sleep metrics, such as total sleep time, sleep efficiency, and wake after sleep onset. Importantly, the vast majority of recordings fell within acceptable thresholds of difference compared to polysomnography, with satisfactory agreement in over 90 per cent of recordings for all of these metrics.

Small systematic biases relative to standard polysomnography were observed. The Muse-S overestimated sleep efficiency, overall sleep duration, deep sleep, and REM sleep while underestimating sleep onset latency and light sleep. The magnitude of these biases was within previously proposed margins for satisfactory differences [9–11]. Although some degree of bias is unavoidable, careful documentation of these biases is necessary to inform the choice of appropriate technologies to be used to address specific research questions and to anticipate the limitations that may restrict interpretations.

The Muse-S showed high accuracy, sensitivity, and specificity, but its performance varied significantly by sleep stage. Substantial to near-perfect agreement was observed for NREM2, NREM3, and REM sleep, as well as wake, but NREM1 sleep remained at the fair to moderate agreement level. This limitation is consistent with prior research on other portable EEG devices, where light sleep stages often pose classification challenges [2]. Of note, NREM1 sleep also shows the poorest agreement rates for human scoring of standard polysomnography [16]. The difficulty in detecting NREM1 sleep likely stems from its transitional nature and the subtle EEG patterns that characterize this stage. NREM1 is often marked by low-amplitude mixed-frequency activity, which can overlap with wakefulness or NREM2. These complexities underscore the importance of refining automated algorithms to improve sensitivity and specificity for light sleep detection. This will be a critical aspect to refine for the characterization of sleep abnormalities in clinical populations.

Some of the Muse-S imprecisions may partly be attributed to the reduced number of EEG channels and reliance on frontal electrode placements. Further advancements of automated pre-processing and sleep stage scoring tools may have to rely on other information contained in the limited portable EEG signals that may co-occur with NREM1 sleep. As with standard EEG recordings, movements do create artifacts in the Muse-S signal. Advances in signal processing may eventually generate refined means of preserving signal quality during movements, which would be especially relevant in clinical groups who have restless nights. Furthermore, in cases of more localized artifacts, future work should explore whether dynamically changing the selected referencing electrode may enable enhancements in signal quality. Since two ancillary channels can be added to the Muse-S, future studies could also assess the relative gains in sleep stage scoring precision resulting from the integration of additional physiological signals, such as occipital EEG, EMG, or EOG.

In our view, it is critical that the refinements and further performance testing of portable EEG devices and their algorithms be based not only on adult good sleepers, but also on diverse samples of individuals from all ages with different sleep profiles and neurological backgrounds. The current study started mapping promising outcomes on this front since 80 per cent of participants had PSQI scores suggestive of poor sleep and 17 per cent had sleep-related breathing disorders. Exploratory subgroup analyses confirmed that there was a similar level of agreement between the Muse-S device and standard polysomnography across different types of sleepers.

Visual signal inspection confirmed that sleep microarchitecture features, such as rapid eye movements, *k*-complexes, spindles, and sleep slow waves were accurately captured by the Muse-S. This positions the Muse-S as a potential tool for investigating graphoelements and neurophysiological sleep processes such as sleep homeostasis in naturalistic environments. Accordingly, subsequent work needs to be done to assess the performance of portable EEG devices like the Muse-S in delineating quantified EEG metrics, from spectral analyses to discrete events detection.

Although the present study was focused on automated scoring of Muse-S data, Muse-S technology offers researchers the access to EEG signals in European Data Format (EDF) allowing the flexibility of scoring sleep manually if desired. The sleep community started mapping adaptations to AASM scoring guidelines to score portable EEG data limited to restricted set of channels (evolving resources and standard operating procedures are available on the Canadian Sleep Research Consortium website: www.researchsleep.ca/researchers).

Signal quality and artifact management are central challenges for portable sleep monitoring in the field. Realistic expectations should take into account that portable EEG is bound to face inherent challenges that are classically circumvented in in-lab polysomnography. In the present study, 16 per cent of the Muse-S recordings were excluded due to poor signal quality, highlighting the need for robust artifact rejection algorithms and improved sensor designs. Refining adaptive filtering techniques and real-time feedback on electrode placement could enhance signal reliability and reduce data loss. Also, the occurrence of data loss and poor signal quality can be minimized by detailed instructions and remote monitoring of ongoing data collection to address technical issues before subsequent nights are recorded. We observed decreased signal quality that seemed to arise due to poor electrode contact with the skin or due to the wear and tear of the Muse-S device after 14 to 20 nights of use. In some cases, applying moisture through a damp cloth or conductive EEG paste on the electrodes of the Muse-S did help improve signal quality. Ongoing optimization methods for data collection in the context of sleep research will facilitate field implementation.

The ability to capture EEG-based sleep metrics in a portable format opens exciting new doors. Unlike single-night polysomnography, which is influenced by first-night effects [17], the Muse-S allows for multi-night data collection in naturalistic settings. This has the potential to expand capacity for assessing night-to-night variability, long term longitudinal monitoring, investigations focused on environmental factors part of the natural sleeping environment, and large-scale population studies. For instance, this type of multi-night in-depth monitoring in the field may unveil novel information about insomnia phenotypes and how they relate to environmental conditioning. In the age of personalized medicine, real-time EEG monitoring has important clinical implications with regards to increased accessibility to physiological phenotyping that may help identify specific sleep profiles and adapted treatment planning. This could also help track detailed changes in sleep physiology across standard treatments, serving as an educational and motivational tool for patients and enabling healthcare providers and scientists to track relevant outcomes and explore potential mechanisms of action. This also sets the ground for the development of novel sleep interventions embedding individual sleep features in real time. However, further work is required to delineate the boundaries of appropriate interpretation in various clinical groups.

This study had some limitations. Firstly, all data were collected in a controlled laboratory environment, where technicians, rather than the participants themselves, installed the Muse-S device. This limits inferences that can be made about the usability of this device by non-specialized users. Secondly, all polysomnography data were scored only once, preventing any assessment of the reliability of human scoring for the gold standard against which the Muse-S data were compared.

In conclusion, this study contributes to the growing body of evidence supporting the performance of portable EEG devices, with clear implications for sleep research and clinical practice. The Muse-S device demonstrates substantial agreement with polysomnography for global sleep metrics and most sleep stages, establishing its strong performance as a portable tool for sleep EEG monitoring. While certain limitations in signal quality and the identification of light sleep remain, ongoing advancements in sensor technology and algorithm development are likely to lead to further improvements. By providing a scalable and accessible alternative to polysomnography, portable EEG devices represent a significant step toward democratizing access to sleep assessments and advancing the understanding of sleep health in diverse populations. Eventually, this type of technology could increase access to assessments of sleep architecture for individuals with time, energy, and financial limitations hindering their ability to visit sleep laboratories. This could also facilitate a more inclusive comprehension of sleep disorders across diverse clinical, demographic, and socioeconomic profiles. Future efforts should focus on refining sleep stages detection in a broader range of sleepers to ensure robustness across age, sex, and sleep pathologies. There is also a need to explore innovative applications of these technologies to maximize their impact on sleep medicine and public health.

## Data Availability

The data underlying this article cannot be shared publicly due to ethical reasons. The data will be shared on reasonable request to the corresponding author.
